# Noninvasive Optical Sensing of Aging and Diet Preferences Using Raman Spectroscopy

**DOI:** 10.1021/acs.analchem.4c05853

**Published:** 2025-01-01

**Authors:** Isaac D. Juárez, Alexandra Naron, Heidi Blank, Michael Polymenis, David W. Threadgill, Regan L. Bailey, Patrick J. Stover, Dmitry Kurouski

**Affiliations:** † Department of Biochemistry and Biophysics, Texas A&M University, College Station, Texas 77843, United States; ‡ Department of Nutrition, Texas A&M University, College Station, Texas 77843, United States; § Institute for Advancing Health through Agriculture Texas A&M University, College Station, Texas 77843, United States

## Abstract

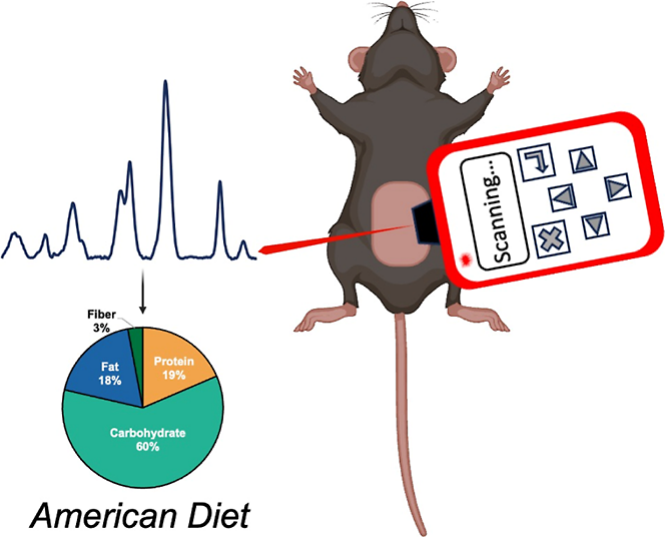

Effective dietary strategies and interventions for monitoring dietary exposures require accurate and noninvasive methods to understand how diet modulates health and risk of obesity; advances in technology are transforming the landscape and enabling more specific tailored approaches to nutritional guidance. This study explores the use of Raman spectroscopy (RS), a noninvasive and nondestructive analytical technique, to identify changes in the mice skin in response to constant dietary exposures. We found that RS is highly accurate to determine body composition as a result of habitual dietary patterns, specifically Vegan, Typical American, and Ketogenic diets, all very common in the US context. RS is based on major differences in the intensities of vibrational bands that originate from collagen. Moreover, RS could be used to predict folate deficiency and identify the sex of the animals. Finally, we found that RS could be used to track the chronological age of the mice. Considering the hand-held nature of the utilized spectrometer, one can expect that RS could be used to monitor and, consequently, personalize effects of diet on the body composition.

## Introduction

Poor dietary quality is the underlying cause of numerous pathologies, including diabetes, cardiovascular disease, and cancer.^1^ The metabolic response to food exposures is further complicated by dissimilar responses to the same food or diets by different individuals or population groups.^2−4^ For instance, consumption of lipid- or carbohydrate-rich foods may cause obesity in some individuals and very little changes in the body weight in others.^5,6^ Thus, it is reasonable to expect that personalized or precision guidance toward optional dietary patterns may reduce the risk of diet-related chronic diseases. This transformative concept allows for optimization of dietary intake for individuals with different genetics, related health behaviors and exposures, and similar metabolic patterns.^7,8^

Given the inherent limitations of self-reported dietary data, objective measures of dietary intakes are helpful to not only understand what was consumed but also how it was metabolically processed and can be used in isolation or in tandem with self-reported data. Objective measures require the development of sensors that can be used to (i) analyze nutritional composition of consumed food and (ii) monitor changes in body biochemistry triggered by the consumed food.^9−11^ Vibrational spectroscopy like Raman spectroscopy (RS) and infrared spectroscopy (IR) have previously been used for the imaging and characterization of biological tissue.^12−14^ Additionally, we previously demonstrated that RS could be used for noninvasive, nondestructive, label-free, and highly accurate quantification of macronutrients in foods.^15^ In the agricultural sector, this technique can also be used to probe the ripeness of fruits and vegetables. Using HPLC, Dhanani showed that the concentration of carotenoids changes during fruit ripeness.^16^ In this case, RS was used to detect changes in the concentration of carotenoids in the fruit rind. RS can also detect and identify changes in plant biochemistry that are caused by fungi, including those that produce aflatoxin, a highly toxic, and carcinogenic substance.^17^

Here, we investigated whether RS data could be used to detect differences in the body composition using data on mice that were fed different diets under controlled conditions. For this, we exposed C57BL/6J (B6) mice to American and Ketogenic diets that represented carbohydrate- and fat-rich diets, respectively. The third group of B6 mice was kept on a Vegan diet, which has low protein and fats. Previously reported results showed that all three diets drastically changed animal biochemistry and gene expression.^18^ After the animals spent 3 months on each dietary pattern, the coat hair from the abdomens was removed. Using a hand-held Raman spectrometer, we acquired spectra from each animal using standardized procedures. Using the same experimental approach, we also analyzed biochemical changes in a genetically diverse outbred mice population (the Simplified Diversity Outbred or SDO) that were exposed to American, Mediterranean, Ketogenic, Japanese, standard, and Vegan diets previously described elsewhere.^18^ Importantly, these mice have a genetic composition and metabolic disease risk as humans.

In addition to body composition, we aimed to investigate other metabolic changes in response to the diet in these mice. Aging changes the biochemical profiles and gene expression in living organisms. Expanding upon this, we asked if RS could be used to predict mice’s chronological age based on the changes in the structure and composition of their skin, namely collagen. Given that one carbon metabolism is tied to both diet and aging, we examined the relationship of RS and biochemical changes in mice caused by the lack of folate, the largest dietary contributor to one carbon in human models.

## Results and Discussion

**Figure 1:**
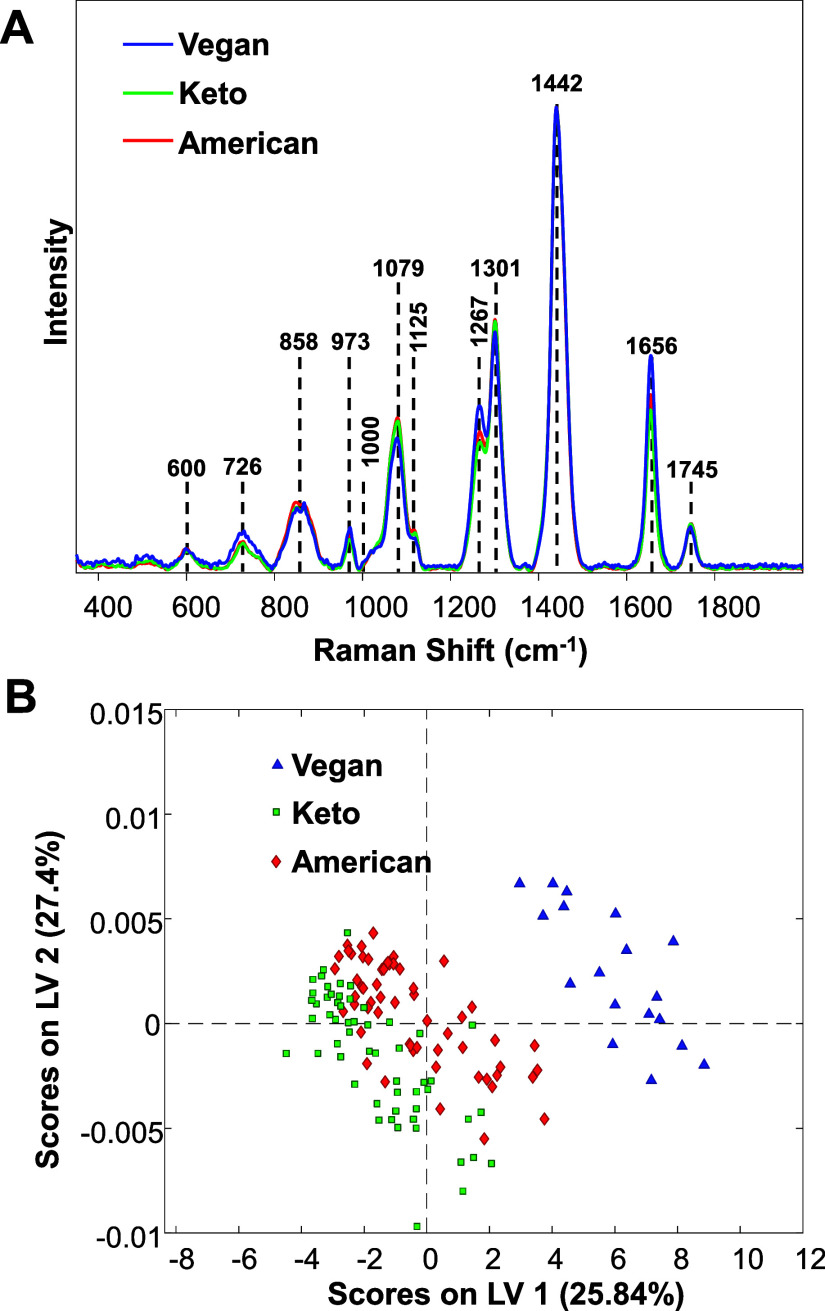
Averaged Raman spectra acquired from mice skin (top) exposed to American, Ketogenic, and Vegan diets with the corresponding latent variable (LV) plot (bottom).

In the acquired Raman spectra, we observed vibrational bands of a polypeptide backbone known as amide I (1656 cm^–1^) and amide III (1267 and 1301 cm^–1^), as well as vibrations that could be assigned to aromatic amino acids (729 and 1000 cm^–1^) of proteins (Figure 1 and Table 1) that correspond to macronutrient distributions in the three dietary exposures. We also found vibrational bands that could be assigned to lipids (1079, 1125, and 1745 cm^–1^) and the peak at 1442 cm^–1^ which originated from CH_2_ vibrations. Because this chemical moiety is present in all classes of biological molecules, we normalized the acquired spectra on this vibrational band.

**Table 1:** Assignment of Vibrational Bands Observed in the Raman Spectra Collected from Mice

band	vibrational mode	assignment
600	ring vibration	cholesterol^19^
729	ring vibration	aromatic amino acids^20^
858	C–C backbone	collagen^21^
973	C–C backbone	collagen^21^
1000	ring vibration	aromatic amino acids
1079	ν(C–C)	lipids^22,23^
1125	ν(C–C)	lipids^22,23^
1267	amide III (C–N stretch)	unsaturated lipids, proteins^20,24^
1301	amide III (C–N stretch)	unsaturated lipids, proteins^20,24^
1442	CH_2_	aliphatic^25^
1656	amide I (C=O)	unsaturated lipids, proteins^20,24^
1745	C=O	lipids^26,27^

We found that Raman spectra acquired from B6 mice exposed to an American diet exhibited weaker intensities of 973, 1267, and 1656 cm^–1^ bands compared to the spectra collected from mice kept on the Ketogenic diet, as shown in Figures 1 and S1. At the same time, the intensities of these bands were stronger in the spectra acquired from mice exposed to a Vegan diet. Based on these results, we can conclude that composition of the diets affects both the amount and the order of collagen in the mouse skin. Based on the relative intensities of 1267 and 1301 cm^–1^ bands, we can conclude that Vegan fed mice had the highest collagen content and Ketogenic fed mice the lowest collagen content in their skin. Vegan fed mice also had the least disordered collagen, whereas the distortions in the collagen structure were the greatest in the mice exposed to the Ketogenic diet. These results indicate that diets alter the collagen structure that could be detected by RS to enable sensing of mouse dietary consumption.

We also found that Raman spectra acquired from mice fed American and Ketogenic diets exhibited the strongest intensities at 1079 and 1125 cm^–1^, which could be associated with the lipid composition of the diets.^22,23^ This indicates that the skin of these animals had much higher amounts of lipids compared to the skin of mice that were fed the Vegan diet. It should be noted that we did not observe substantial differences in the intensities of 1745 cm^–1^ among all acquired spectra. Based on these results, we can conclude that RS could be used to identify changes in the lipid profile of the skin, which, in turn, could be used to predict diet consumption of the animals.

Next, we used PLS-DA to determine the accuracy of Raman-based identification of dietary consumption, as shown in Table 2. We found that habitual American and Ketogenic dietary patterns could be identified with 87.9% and 87.5% accuracies, respectively, whereas Vegan diet could be identified with 100% accuracy.

**Table 2:** Accuracy of Classification by PLS-DA for Spectra Acquired from Mice Exposed to American, Keto, and Vegan Diets

predicted as	accuracy (%)	American	Keto	Vegan
American	87.9	51	7	0
Keto	87.5	7	49	0
Vegan	100	0	0	20

**Figure 2:**
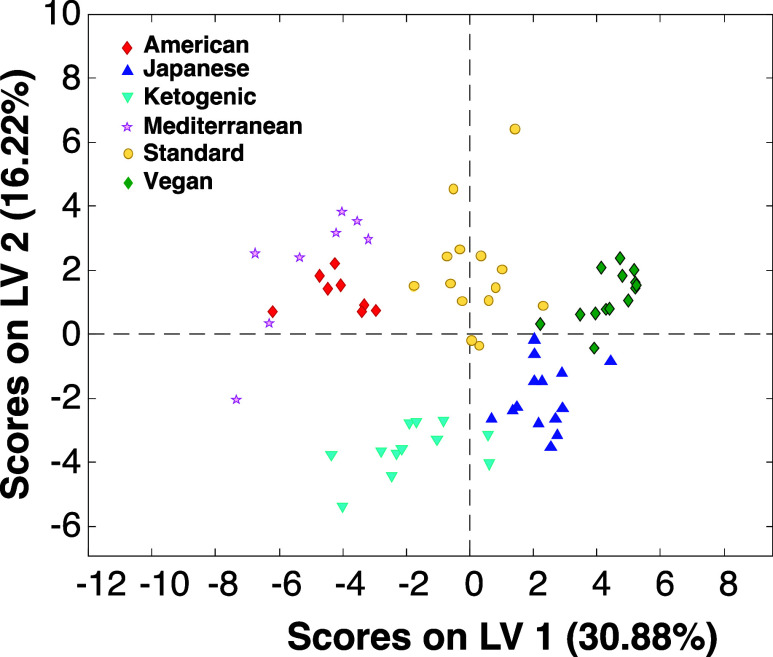
LV plot shows clustering of Raman spectra acquired from the skin of sacrificed mice exposed to American, Mediterranean, Ketogenic, Japanese, standard, and Vegan diets.

Expanding upon this, we investigated the extent to which RS could be used in a genetically diverse mouse population (SDO mice) to differentiate between six different diets: American, Mediterranean, Ketogenic, Japanese, standard, and Vegan diets; as seen in Figure S2. PLS-DA analysis revealed on average ∼80% accuracy in the identification of the diet consumption of mice if the spectra were acquired from live animals (Table 3) or the skin of sacrificed ones (Table 4 and Figure 2). These results indicate that diet-specific changes in the structure and composition of skin can be used to track dietary consumption.

**Table 3:** Accuracy of Classification by PLS-DA for Spectra Acquired from Mice Exposed to American, Mediterranean, Ketogenic, Japanese, Standard, and Vegan Diets

predicted as	accuracy (%)	American	Japanese	Keto	Mediterranean	standard	Vegan
American	100	6	0	1	0	0	0
Japanese	80	0	4	0	1	0	0
Keto	66.6	0	1	4	0	1	1
Mediterranean	80	0	0	1	4	0	0
standard	87.5	0	0	0	0	0	0
Vegan	80.0	0	0	0	0	0	4

**Table 4:** Accuracy of Classification by PLS-DA for Spectra Acquired from the Skin of Sacrificed Mice Exposed to American, Mediterranean, Keto, Japanese, Standard, and Vegan Diets

predicted as	accuracy (%)	American	Japanese	Keto	Mediterranean	standard	Vegan
American	75.0	6	1	0	3	1	0
Japanese	78.6	0	11	1	0	0	0
Keto	91.7	0	2	11	0	0	0
Mediterranean	62.5	0	0	0	5	0	0
standard	92.9	2	0	0	0	13	0
Vegan	100	0	0	0	0	0	14

**Figure 3:**
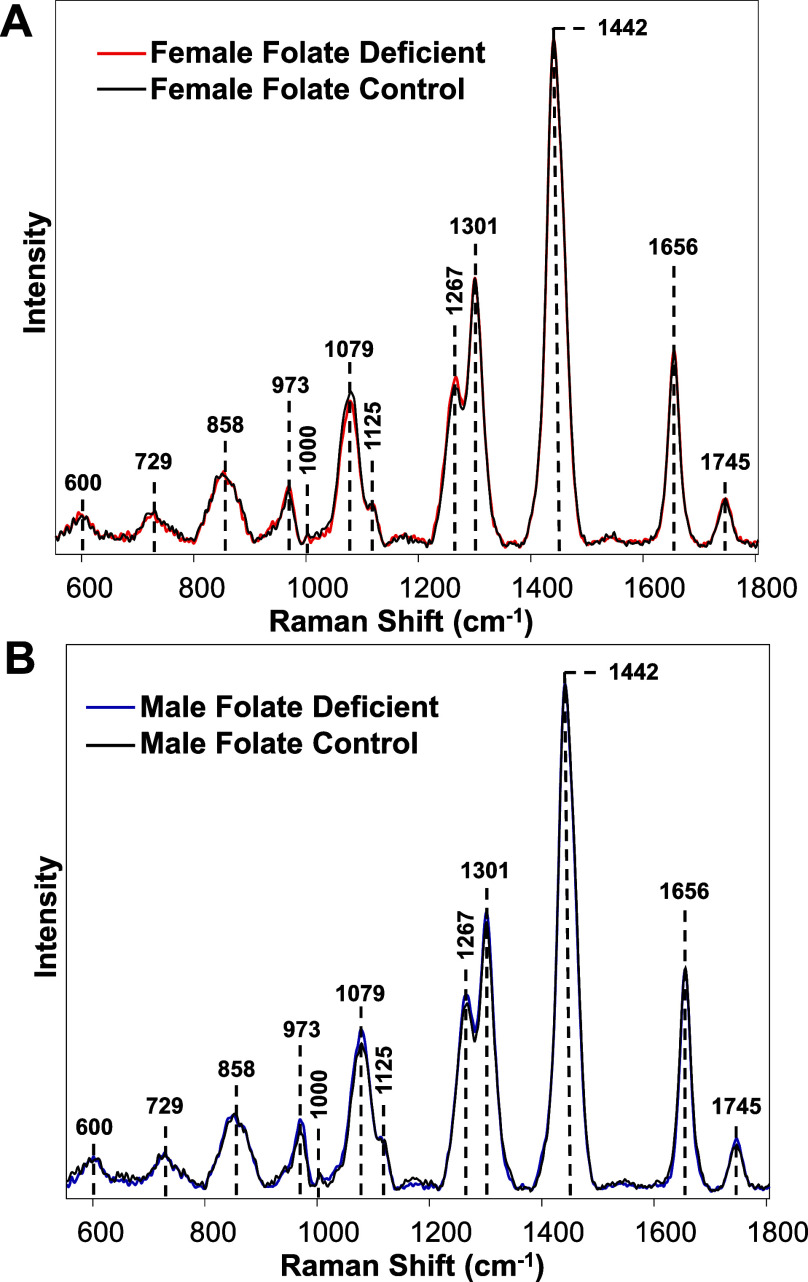
Averaged Raman spectra acquired from the skin of female (A) and male (B) mice that were kept on folate-free diet for 12 months and control animals.

We next asked whether RS could be used to predict the folate deficiency. This vitamin prevents congenital abnormalities by regulating cell growth and division.^28^ Although the effects of low folate levels in diets are poorly understood; recently, Blank and co-workers demonstrated that B6 mice exposed to folate-free diets had decreased anabolic biosynthetic processes and enhanced metabolic plasticity.^28^ The researchers also observed that changes induced by the absence of folate were different in male vs female mice. Expanding upon this, we acquired spectra from the skin of ∼100 week old male and female mice that were kept for 12 months on a folate-free diet as well as from the skin of animals on the standard folate-replete diet (control), Figure 3.

We found that the intensity of the 1267 cm^–1^ band in the spectra acquired from both male and female mice exposed to a diet lacking folate was greater than in the spectra acquired from the skin of control animals on a folate-replete diet. These results indicate that lower folate helps to minimize collagen distortion in the animal skin. We also found that in the spectra acquired from male mice exposed to the folate-free diet, the intensity of 1079 cm^–1^ band, which could be assigned to lipids,^22,23^ was greater than in the spectra acquired from control male animals. These results indicate that a folate-free diet facilitates lipid accumulation in male mice. However, the opposite behavior of this band was observed in the female mice. Specifically, the intensity of 1079 cm^–1^ peak was slightly stronger in the spectra acquired from control mice compared to the animals kept on a folate-deficient diet. These results indicate that in female mice, the absence of folate in the diet lowers the rate of lipid accumulation in the derma. PLS-DA revealed that absence of folate in the diet could be predicted with 82.4% in male and 84.8% in female mice, Table 5.

**Table 5:** Accuracy of Classification by PLS-DA for Spectra Acquired from Male and Female Mice Exposed to Folate-Free Diet and Control Animals

	male (%)	female (%)
folate complete	79.1	70.4
folate deficient	82.4	84.8

**Figure 4:**
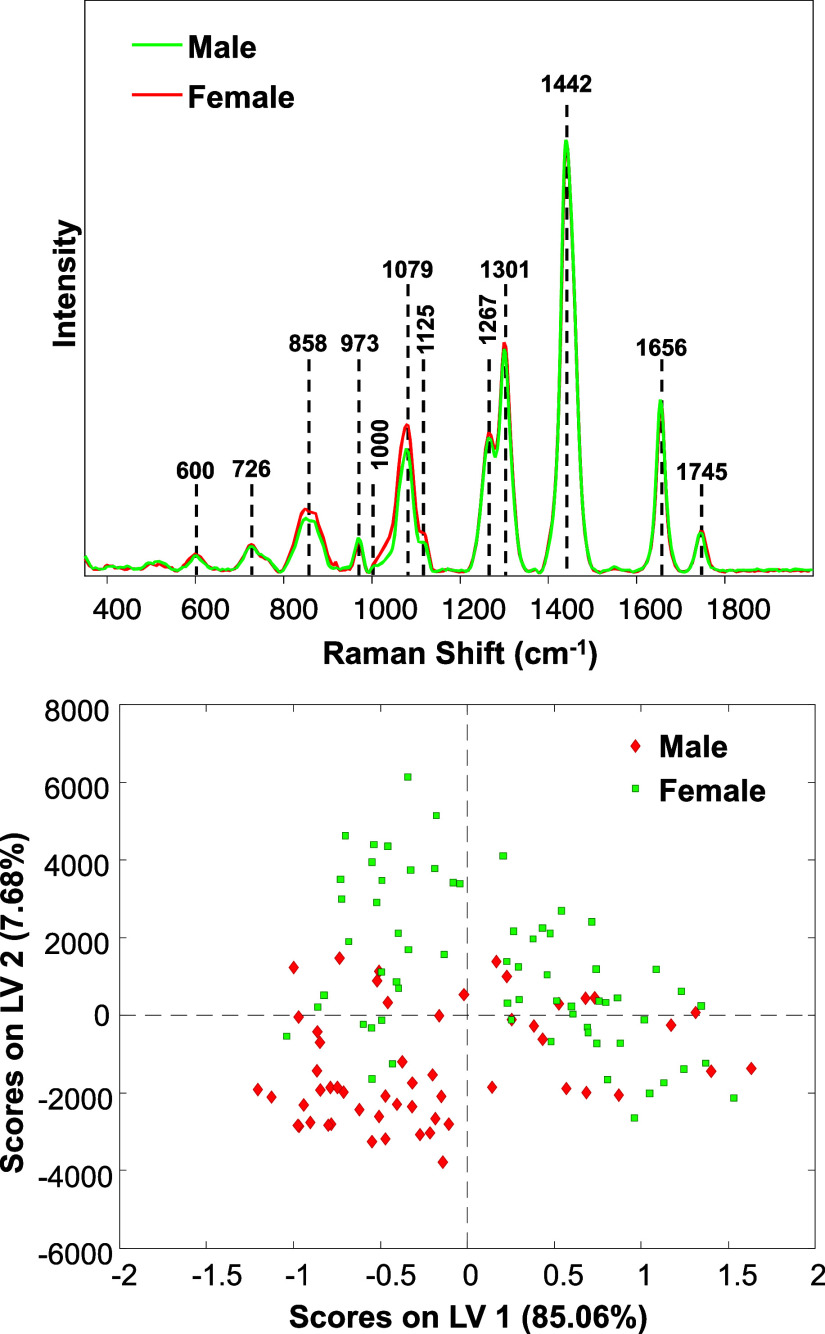
Averaged Raman spectra acquired from male and female mice (top) with the corresponding LV plot (bottom).

Expanding upon these findings, we investigated whether RS could be used to differentiate between sexes of mice. Our results show that vibrational signatures of male and female mice are substantially different, as shown in Figure 4.

Specifically, we found that Raman spectra acquired from female mice exhibited a much greater intensity of 1079 and 1125 cm^–1^ bands compared to the spectra acquired from male mice. This indicates that female mice possess substantially greater amount of skin fat compared to male animals, which is consistent with what has been reported in humans where females have higher levels of subcutaneous fat.^29^ We also observed stronger intensity of 858, 1267, and 1301 cm^–1^ bands in the spectra acquired from female compared to male animals, suggesting that these animals have slightly higher order of collagen in their skin.

PLS-DA showed that RS could be used to enable highly accurate differentiation between the sex of mice, as listed in Table 6. We found that female and male sex could be identified with 87.3% and 92.3% accuracies, respectively.

**Table 6:** Accuracy of Classification by PLS-DA for Spectra Acquired from Female and Male Mice

predicted as	accuracy (%)	female	male
female	87.3	48	5
male	92.3	7	60

**Figure 5:**
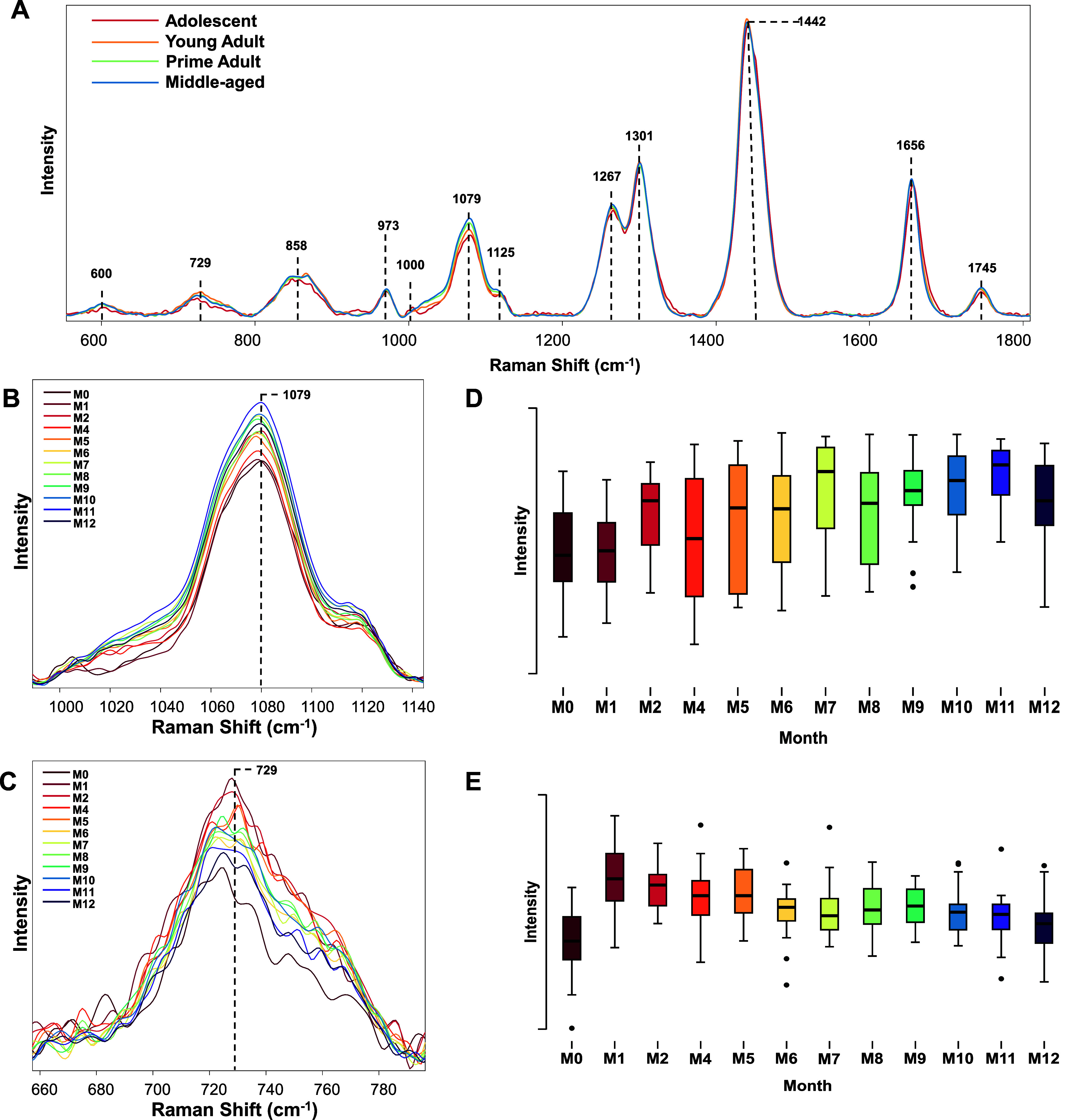
(A) Averaged Raman spectra acquired from adolescent (M0), young adult (M1–M5), prime adults (M6–M8), and middle-aged (M9–M12). Changes in the intensity of 1079 (B) and 729 cm^–1^ (C) with the corresponding ANOVA graphs (D and E, respectively). According to one-way ANOVA, *P < 0.05, **P < 0.01, and ***P < 0.001.

Next, we investigated the extent to which RS could be used to track the age of the mice. For this, RS were acquired every two months from mice kept on an American diet, as shown in Figure 5 and Table 7. In this study, month zero (M0) corresponds to the 1 month old animals just exposed to the American diet, month 1 (M1), 2 month-old animals exposed to the American diet for 1 month, etc. We observed a gradual increase in the intensity of 1079 cm^–1^ band, which could be assigned to lipids, in the acquired spectra as the age of animals increased. These results indicate that animals accumulate more fat in their derma as they age, similar to humans.

**Table 7:**
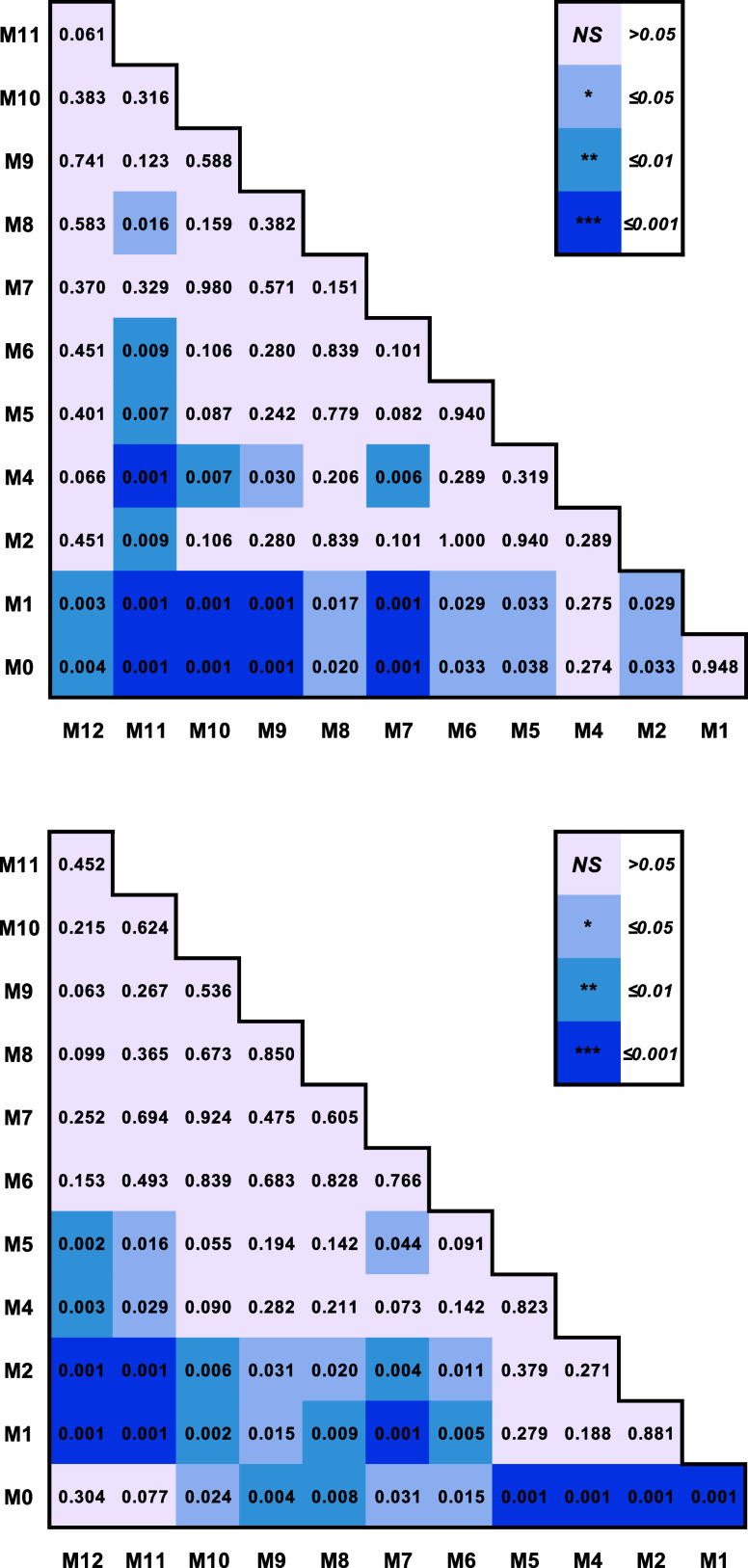
ANOVA of Spectroscopic Changes in 1079 (Top) and 729 (Bottom) Peaks Acquired from the Spectra of Mice at M0–M11

We also observed substantial changes in the intensity of 729 cm^–1^ (associated with aromatic amino acids) in the spectra acquired from M0 and M1 animals. This initial increase in the peak intensity was followed by a gradual decrease in the intensity of the 729 cm^–1^ band as the age of the animals increased, as shown in Figure 5. These changes point to the structural rearrangements associated with (i) exposure to different diets and (ii) age-related changes in the structure of collagen in the skin.

Finally, we utilized PLS-DA to measure the accuracy with which different age groups of mice could be predicted, as shown in Table 8. We found that adolescence (M0) could be identified with 100% accuracy, whereas young (M1–M5) and prime (M6–M8) ages could be correctly identified with 77.1 and 63.8% accuracy, respectively. PLS-DA also indicated that middle-age (M9–M12) could be correctly predicted using RS. These results demonstrate that RS coupled with PLS-DA could be used to predict the chronological age of animals. Our results also indicate that age-sensing is based on the changes in the amount of fats in the skin and on the secondary structure of collagen that is altered during aging.

**Table 8:** Accuracy of Classification by PLS-DA for Spectra Acquired from Mice at Adolescent (M0), Young Adult (M1–M5), Prime Adults (M6–M8), and Middle-Aged (M9–M12) Stages

predicted as	accuracy (%)	adolescent (*N* = 19)	young adult (*N* = 83)	prime adult (*N* = 58)	middle-aged (*N* = 80)
adolescent	100	19	0	0	0
young adult	77.1	0	64	14	5
prime adult	63.8	0	14	37	12
middle-aged	78.8	0	5	7	63

Altogether, these results highlight the versatility and effectiveness of RS in detecting metabolic changes in live animals. However, some limitations warrant further study. Peak assignments in RS remain dynamic and therefore so does peak interpretation. Additionally, the findings of this study are specific to the carefully modeled diets used, particularly regarding the use of RS for determining chronological age. As such, different outcomes may arise in mice with varied diets or greater genetic diversity. Future research should focus on exploring RS in genetically diverse mice and investigating the potential of IR spectroscopy for similar applications.

## Conclusions

Our results show that diets alter the chemical structure, composition, and integrity of collagen in the skin. These chemical changes could be sensed using RS. Consequently, Raman spectra acquired from the skin could be used to predict the diets consumption of animals. Our results also indicate that male and female mice have small differences in the chemical composition of their skin, which allows for RS-based identification of animal sex. Finally, a 12 month study revealed that the concentration of fat increased in the skin with aging. We also observed age-related changes in the structure of the collagen. These changes could be tracked using RS, enabling prediction of the chronological age of animals. These findings indicate that hand-held Raman spectrometers and, ultimately, wearable devices could be used to personalize diet and track metabolic changes associated with aging and potentially vitamin-deficiencies.

## Experimental Section

### Mice

The human relevant American, Japanese, Ketogenic, Mediterranean, Vegan, and standard have been described elsewhere.^18^ In addition to B6 mice, a genetically diverse outbred mouse population was used that will be described elsewhere.^18^ Briefly, an outbred population was generated by random mating of the wild-derived strains CAST/EiJ, PWK/EiJ, and WSB/EiJ to produce a SDO population. The diets and B6 mice used in Figure 3, exposed to folate-limited and-replete diets (Table S1), were from a study published recently, and have been described elsewhere.^28^

### Raman Spectroscopy

Prior to the spectral acquisition, the undersides of all mice were depilated with Nair. An Agilent Resolve hand-held Raman spectrophotometer was then used to collect spectra from each mouse’s abdomen at 830 nm. This wavelength was chosen for a deeper penetration and to avoid a strong background signal caused by autofluorescence at shorter excitation wavelengths. Acquisition time was 1 s at a laser power of 495 milliwatts. No spatial offset was used.

Maximum permissible exposure for the laser was calculated to be 20,017 J/m^2^, while the actual exposure used was calculated as 39,391 J/m^2^. Nevertheless, all mice were monitored during scanning, and no burn damage or unusual behavior was evident. Twenty Raman spectra were acquired for each group of mice, at different intervals for each experiment. All spectra were baselined automatically by the Resolve software and normalized at the 1440 cm^–1^ peak.

### Chemometrics

PLS_toolbox (eigenvector Research Inc.) was used in MATLAB to perform all statistical analyses and to create all figures. Data was downloaded from the instrument as CSV files then imported into MATLAB. Kruskal–Wallis ANOVA and Dunn’s test was performed for all peaks with a visual change. PLS-DA models were built for comparison of each experimental group, with 6 to 11 LVs used for each model, as shown in Table S2.

## Supporting Information Available

2D-COS analysis, Raman spectra, number of latent variables, and description of mice utilized in each experiment (PDF)
