# Supplementary material for: Noninvasive Optical Sensing of Aging and Diet Preferences Using Raman Spectroscopy
Source: Anal Chem. 2025 Jan 1;97(1):969–75. doi: 10.1021/acs.analchem.4c05853 (PMC11740184; doi:10.1021/acs.analchem.4c05853)
Supplement: Supplementary file 1 — ac4c05853_si_001.pdf [file ac4c05853_si_001.pdf]

Supporting Information for "Non-Invasive Optical Sensing of Aging and Diet Preferences Using Raman Spectroscopy"

Isaac D. Juarez[1], Alexandra Naron[1], Heidi Blank[1], Michael Polymenis[1], David W. Threadgill[1], Regan L. Bailey,[2,3] Patrick J. Stover[1,3]*, Dmitry Kurouski[1,3]*

[1]Department of Biochemistry and Biophysics, Texas A&M University, College Station, Texas 77843, United States
[2]Department of Nutrition, Texas A&M University, College Station, Texas 77843, United States
[3]Institute for Advancing Health through Agriculture Texas A&M University, College Station, Texas 77843, United States

Supporting Information

Table S1. Description of mice utilized in each experiment.

Experiment	Mouse Strain	Diet	Number
Global Diet	SDO	American	7F
		Japanese	8F
		Ketogenic	8F
		Mediterranean	8F
		Standard	9F
		Vegan	8F
High Carb, High Fat	C57BL6/J	American	10M
		Ketogenic	10M
Folate Deficiency	C57BL6/J	Folate Complete	6F, 12M
		Folate Deficient	8F, 13M
Aging	C57BL6/J	Vegan	10M

Table S2. Number of latent variables utilized to build each model.

Model	Latent Variables
Table 2	11
Table 3	9
Table 4	11
Table 5	10

Table 6	8
Table 8	6

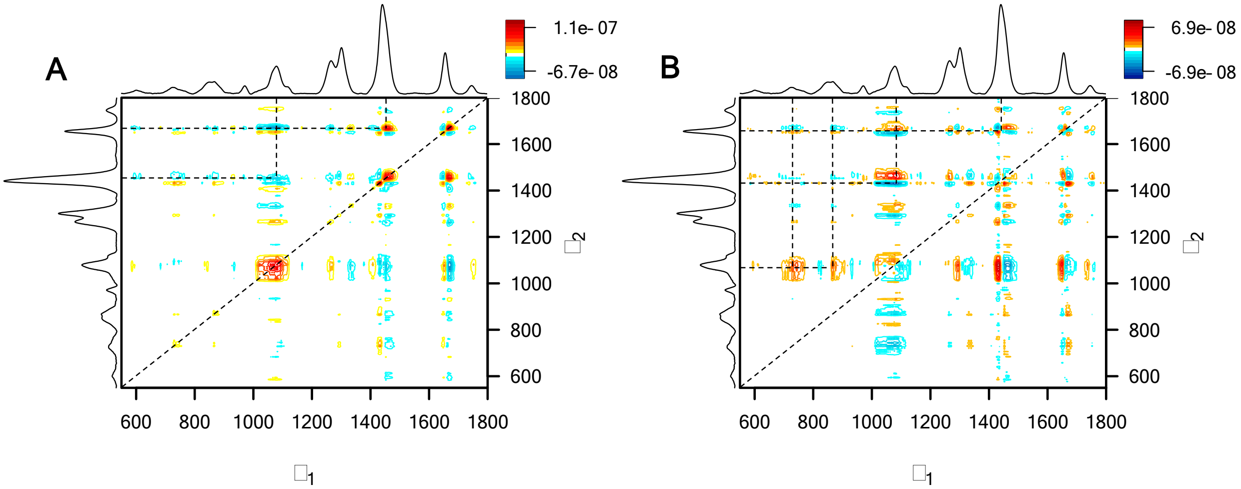

Figure S1. 2-D correlation spectroscopy (2D-COS) analysis of the aged vegan mice spectra. (A) Synchronous 2D correlation spectrum depicts peaks that change together, while (B) asynchronous 2D correlation spectrum depicts the order of peak changes.

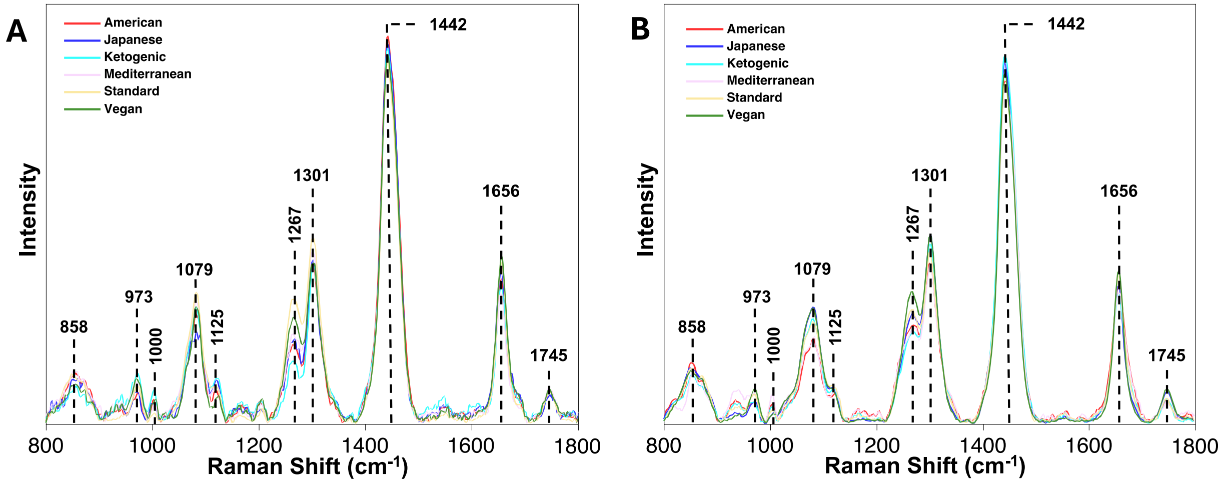

Figure S2. Spectra acquired from (A) live mice and the (B) skin of sacrificed mice exposed to American, Mediterranean, Keto, Japanese, Standard, and Vegan diets.
